# Variables associated with successful vascular access cannulation in hemodialysis patients: a prospective cohort study

**DOI:** 10.1186/s12882-019-1373-3

**Published:** 2019-05-31

**Authors:** Linda L. Coventry, Jon M. Hosking, Doris T. Chan, Evelyn Coral, Wai H. Lim, Amanda Towell-Barnard, Diane E. Twigg, Claire M. Rickard

**Affiliations:** 1 Centre for Nursing Research, Sir Charles Gairdner Hospital, Hospital Avenue, Nedlands, Western Australia 6009; 2 School of Nursing & Midwifery, Edith Cowan University, 270 Joondalup Drive, Joondalup, Western Australia 6027; 3 Diaverum Toto Ora Dialysis Clinic, 10 Waddon Place, Mangere, New Zealand; 4 Department of Renal Medicine, Sir Charles Gairdner Hospital, Hospital Avenue, Nedlands, Western Australia 6009; 5 Alliance for Vascular Access Teaching and Research, Menzies Health Institute Queensland and School of Nursing and Midwifery, Griffith University, Nathan Campus, 170 Kessels Road, Nathan, Queensland 4111 Australia

**Keywords:** Arteriovenous fistulae, Cannulation, Cannulation-related complications, Catheterization, Hemodialysis, Nursing, Renal dialysis, Vascular access

## Abstract

**Background:**

Successful vascular access (VA) cannulation is integral to the delivery of adequate dialysis, highlighting the importance of ensuring the viability of arteriovenous access in hemodialysis (HD) patients. Missed VA cannulation can lead to infection, infiltration, hematoma or aneurysm formation resulting in the need for access revision, central venous catheter (CVC) placement, or permanent loss of VA. Cannulation-related complications can also negatively impact on a patient’s dialysis experience and quality of life. This study aimed to identify patient, VA and nurse factors associated with unsuccessful VA cannulations.

**Methods:**

A prospective cohort study was conducted in HD patients with a permanent VA from three HD units. Data on patient, VA and nurse characteristics, plus, cannulation technique were collected for each episode of cannulation. General Estimating Equation was used to fit a repeated measures logistic regression to determine the odds of cannulation success.

**Results:**

We collected data on 1946 episodes of cannulation (83.9% fistula) in 149 patients by 63 nurses. Cannulation included use of tourniquet (62.9%), ultrasound (4.1%) and was by rope ladder (73.8%) or area (24.7%) technique. The miscannulation rate was 4.4% (*n* = 85) with a third of patients (*n* = 47) having at least one episode of miscannulation. Extravasation (*n* = 17, 0.9%) and use of an existing CVC (*n* = 6, 0.6%) were rare. Multivariable characteristics of successful cannulation included fistula compared with graft [OR 4.38; 95%CI, 1.89–10.1]; older access [OR 1.68; 95%CI, 1.32–2.14]; absence of stent [OR 3.37; 95%CI, 1.39–8.19]; no ultrasound [OR 13.7; 95%CI, 6.52–28.6]; no tourniquet [OR 2.32; 95%CI, 1.15–4.66]; and lack of post graduate certificate in renal nursing [OR 2.27; 95%CI, 1.31–3.93].

**Conclusion:**

This study demonstrated a low rate of miscannulation. Further research is required on ultrasound-guided cannulation. Identifying variables associated with successful cannulation may be used to develop a VA cannulation complexity instrument that could be utilised to match to the cannulation skill of a competency-assessed nurse, thereby minimising the risk of missed cannulation and trauma.

## Background

Patients on maintenance hemodialysis (HD) require a well-functioning vascular access (VA) to achieve effective therapy. Vascular access can take the form of an arteriovenous fistula (AVF), arteriovenous graft (AVG) or central venous catheter (CVC). Current clinical practice guidelines [1, 2] recommend the use of an AVF as the preferred VA for HD given its low risk of complications and excellent long-term patency rates. Unfortunately, the creation and maintenance of an AVF continues to be a challenge and remains an important source of morbidity in HD patients [3, 4].

Hemodialysis therapy requires the insertion of arterial and venous needles into the VA. Current literature suggests that cannulation-related complications are an underestimated problem that may seriously affect the outcome of the access [3, 4]. Repeated missed cannulation of either a fistula or graft may result in serious complications such as hematoma, [3–6] infection, [1, 3–5, 7–9] and aneurysm formation [3–9] leading to a need for access revision [3, 7, 10], CVC placement, [5] or loss of access [3, 5, 6, 8]. Furthermore, repeated miscannulation can be painful, result in fear, anxiety and be burdensome for the patient [3, 5, 6, 11, 12].

For both in-centre and satellite dialysis units, nurses or technicians are the primary cannulators. The process of access cannulation involves several steps and can include use of ultrasound to guide needle insertion, use of a tourniquet, and/or application of local anaesthetic (topical, subcutaneous or none). Different techniques are utilised and can include rope ladder, area, or buttonhole cannulation [9, 13, 14]. Rope ladder involves changing the needle placement site for each dialysis session, and choosing a site at a defined distance from the previous site along the VA [9, 13, 14]. Area technique, also known as “one-site-itis” is insertion of the needles in the same general area, session after session; and buttonhole is insertion of the needle in exactly the same site [9, 13, 14]. Nurses also decide on the access needle gauge and its orientation (i.e. whether needle is inserted retrograde or antegrade, bevel up or down), and whether the needle is rotated after insertion.

Most studies [3, 8, 15–20] in the literature examine VA failure, that is, time from access creation to permanent failure. The two studies, [3, 13] which examined cannulation success reported that missed cannulation was associated with the presence of an AVF compared with AVG, [3] limited length of cannulation route, [3] use of back-eye needles, [13] rope-ladder technique, [13] insertion of venous needle first, [13] rotation of arterial needle, [13] and use of 16–17 gauge needles [13].

There is a paucity of data on variables, which influence successful cannulation [5]. As such, there is also a lack of clinical guideline and recommendations on cannulation technique, [21] and evidence to inform best practice [1, 13, 14]. To minimise miscannulation and prevent access related complications, further research is required to determine the various patient, VA and nurse-related factors that are associated with miscannulation. Therefore, this study aimed to identify patient, VA and nurse factors associated with unsuccessful VA cannulations.

## Methods

### Aim, design and setting

The aim of this study is to identify patient, VA and nurse factors associated with unsuccessful VA cannulations. This prospective cohort study was conducted in one in-centre (Sir Charles Gairdner Hospital) and two satellite hemodialysis units (Joondalup Health Campus and Diaverum Stirling Dialysis) in Perth, Western Australia from July 2015 to January 2016. The participants were HD patients with a VA (AVF or AVG) and the HD nurses who were responsible for cannulating the access.

Human research ethics approval was obtained from the study sites and the project team’s university (Sir Charles Gairdner Osborne Park Health Care Group, HREC No: 2015–049; Joondalup Health Campus, HREC No: 1513; and Edith Cowan University, HREC No. 13153). The ethics committee’s approved this low risk study to obtain written informed consent from the hemodialysis nurses and provide the hemodialysis patients with an information sheet and opt-out consent.

### Data collection

A research nurse collected patient and nurse demographic data at study entry. The patient data included demographics, comorbidities, and concomitant medications collected from the patient chart. Patient VA data (collected with consultation from HD nurse) included:

- Age of access
- Type of access (AVF or AVG)
- Surgical revision in the last 3 months
- VA - straight or zigzag
- VA - bifurcation present (nil, single, multiple)
- VA - areas of aneurysm present (yes, no)
- VA - depth of the access (superficial, palpable, non-palpable).

The HD nurse clinical judgement was used to classify the above variables. The nurse data included demographics, work history, education and HD training experience collected via written survey.

Data on episodes of cannulation were collected if both the patient and the nurse agreed to participate in the study. A standardised Case Report Form (CRF) was used to collect data consisting of:

- Prior to cannulation nurse confidence with successful cannulation was collected (scale from 0 not confident to 10 completely confident).
- Length of viable vessel
- Current stenosis
- Bruit (high pitch) indicating area(s) of stenosis
- Stent in usable section of AVF
- Oedema, bruising, hematoma present
- AVF very ‘soft’ with tendency for extravasation
- Tourniquet use
- Ultrasound use
- Rope ladder or area cannulation (no buttonhole cannulation method was included in the study, as it is rarely used at our sites)
- Standard needle length
- Arterial and venous needle gauge
- Arterial needle antegrade or retrograde
- Arterial and venous needle bevel up or down
- Arterial and venous needle rotated after insertion

All patients received conventional HD or haemodiafiltration (HDF) defined as three sessions per week, for four to five hours per session. For all units, the target blood flow rate was 300 to 350 ml/min with a dialysate flow rate of 500 ml/min.

### Outcomes

Cannulation episode success was defined as insertion of two needles (arterial and venous) for HD without extra attempts. Miscannulation was defined as the need to insert more than one needle per arterial or venous connection [3]. Cannulation-related complications included extravasation, hematoma formation, single needle dialysis, use of temporary CVC or abandoning the dialysis procedure. Dialysis adequacy per session was obtained via the online clearance monitoring (Kt/V).

### Statistical analyses

The sample size calculation was based on assuming independent observations, and following the guidelines set out by Peduzzi et al. [22] where p represents the proportion of the population with a failed cannulation attempt (0.04) and k the number of covariates considered (7) in the logistic regression model. Thus, the minimum required number of cannulations required was 1750. However, as patients receive HD three times per week, we cannot assume independence of cannulations. The extent to which this would impact on the required number of individuals for this study was unclear. We therefore collected data from an exhaustive number of patients from the three sites over the study period.

Summary statistics were expressed as mean and standard deviation (SD) for all continuous variables and frequencies and percentages for all categorical variables. Age of access and nurse confidence with successful cannulation were log transformed as they were skewed. Univariable and multivariable generalised estimating equation were used to fit a repeated measures logistic regression model to determine variables associated with first-time cannulation success. Models were conducted separately for patient data, case report data, and nurse data, using repeated measures for patient and nurse identifiers. For nurse variables, we considered those that reflected greater experience or education, which may have affected cannulation competence. All variables with *p* < 0.10 in univariable analyses were included in the multivariable model. This model was simplified in a stepwise fashion by removing the variable with the highest *p*-value and refitting the model until only variables with *p*-values of < 0.05 were retained. As a final check, all excluded variables were retested one at a time in this multivariate model. Odds ratios (OR) and 95% confidence intervals (CI) were provided. Data were analysed using SPSS version 24 (IBM Corp. Released 2015. IBM SPSS Statistics for Windows, Version 22.0 Armonk, NY: IBM Corp).

## Results

During the seven month study period, there were 2071 episodes of VA cannulation in 149 HD patients, performed by 63 dialysis nurses. The average number of episodes of cannulation per patient was 13 (SD 8.8).

### Patient vascular access

The patient (*n* = 149) clinical characteristics, co-morbidities and medications are listed in Table 1. The majority of the dialysis patients were male (63.8%) with a mean age of 68.3 (SD 14.7) years and a mean body mass index of 27.3 kg/m^2^ (SD 6.1). Many of the patients had co-morbidities of hypertension (75.2%), diabetes (54.4%), heart disease (48.3%) and/or peripheral vascular disease (22.8%). Only a small proportion of patients were prescribed anticoagulants (8.7%), platelet aggregation inhibitors (8.1%), immunosuppressant medications (3.4%), and steroids (2.7%).

**Table 1:** Patient, vascular access and nurse characteristics

Patient variables	Mean (SD)	Range	
Age (years)	68.3 (14.7)	30.1–90.9	
BMI (kg/m^2^)	27.3 (6.1)	14.4–64.7	
		n	(%)
Sex	Female	54	(36.2)
	Male	95	(63.8)
Co-morbidities	Diabetes	81	(54.4)
	Peripheral vascular disease	34	(22.8)
	Heart disease	72	(48.3)
	Hypertension	112	(75.2)
	Hypotension	9	(6.0)
	Smoker	7	(4.7)
Medications	Steroids	4	(2.7)
	Immunosuppressant	5	(3.4)
	Anticoagulant	13	(8.7)
	PAI	12	(8.1)
	Mean (SD)	Range	
Age of access (years)	3.8 (4.0)	0.7–29.1	
	Median	Interquartile range	
	2.4	1.6–5.2	
		n	(%)
Type of Access	Fistula	133	(89.3)
	Graft	16	(10.7)
AVF vessel	Brachio-cephalic	78	(58.6)
	Radio-cephalic	47	(35.3)
	Brachio-basilic	6	(4.5)
AVG location	Upper arm	13	(81.2)
	Lower arm	2	(12.5)
	Thigh	1	(6.2)
Access revised in last 3 months	Yes	14	(9.4)
	No	120	(80.5)
AVF Vessel	Straight	95	(63.8)
	Zig-zag	39	(26.2)
Bifurcation	Nil	84	(56.4)
	Single	44	(29.5)
	Multiple	14	(9.4)
Aneurysm	Yes	44	(29.5)
	No	98	(65.8)
Depth of Access	Superficial	55	(36.9)
	Palpable	73	(49.0)
	Non-palpable	1	(0.7)
Nurse characteristics	Mean (SD)	Range	
Age	41.4 (9.8)	20–62	
Years as Registered Nurse	16.2 (9.8)	1–40	
Years as Hemodialysis nurse	9.8 (6.7)	0.4–30	
		n	(%)
Employment Status	Full time	31	(49.2)
	Part time / Casual	31	(49.2)
Sex	Female	53	(84.1)
	Male	10	(15.9)
Highest Level of Education	RN / BN	47	(74.6)
	Postgraduate	15	(23.8)
Job Title	RN	49	(77.8)
	CN / SDN	13	(20.6)
Post graduate in renal nursing	Yes	20	(31.7)
	No	43	(68.3)

*RN* Registered Nurse, *BN* Bachelor of Nursing, *CN* Clinical Nurse, *SDN* Staff Development Nurse, *BMI* Body mass index, *PAI* Platelet aggregation inhibitor, *AVF* Arterio-venous fistula

The AVF was the predominant access (89.3%) and the median age of the access was 2.4 years (interquartile range 1.6–5.2 years). The AVF’s were brachio-cephalic (*n* = 78, 58.6%) and radio-cephalic (*n* = 47, 35.3%) and the majority of the AVG’s were located in the upper arm (*n* = 13, 81.2%). The majority of the VA had not been revised in the previous 3 months (80.5%), the vessel was mostly straight (63.8%), without bifurcation (56.4%), without aneurysm (65.8%), and palpable in nearly half of the patients (49.0%). (Table 1).

### Nurse characteristics

The HD nurses (*n* = 63) were mostly female (84.1%) with a mean age of 41.4 (SD 9.8) years and half (49.2%) working full-time. The majority were Registered Nurses (77.8%) with a mean of 16.2 (SD 9.8) years nursing experience, and a mean of 9.8 (SD 6.7) years as a hemodialysis nurse. Nearly a quarter (23.8%) had a postgraduate degree with 31.7% obtaining a postgraduate certificate in renal nursing. (Table 1).

### Cannulation episodes

The episodes of cannulation are summarised in Table 2. Prior to cannulation, the average nurse confidence with successful cannulation was rated 8.6 out of 10. The nurses reported the mean VA length was 12.1 cm, with minimal stenosis (9.0%), bruit (9.7%), bruising (12.9%), and with a stent (3.5%). The majority of nurses used a tourniquet (62.9%) with topical anaesthetic applied (55.8%) and a rope ladder technique (73.8%). An ultrasound machine was rarely used (4.1%) to assist with cannulation, this was consistent with usual practice in the study sites. (Table 2).

**Table 2:** Episodes of cannulation

Variable	Mean (SD)	Range
Nurse confidence with success before cannulation (0–10)	8.6 (2.2)	0–10
Vascular access length (cm)	12.1 (3.7)	2–30
	n	(%)
Stenosis	175	(9.0)
Bruit	189	(9.7)
Stent in useable section of fistula	69	(3.5)
Oedema	59	(3.0)
Bruising	251	(12.9)
Hematoma present	64	(3.3)
AVF soft with tendency to blow	312	(16.0)
Tourniquet used	1225	(62.9)
Ultrasound used	80	(4.1)
Cannulation technique		
Rope ladder	1436	(73.8)
Area	481	(25.0)
Anaesthetic		
None	546	(28.1)
Topical	1085	(55.8)
Subcutaneous	287	(14.7)
Both	28	(1.4)
Standard needle length	1875	(96.4)
Arterial needle retrograde	1523	(78.3)
Arterial needle bevel up	1587	(81.6)
Arterial needle rotated after insertion	824	(42.3)
Venous needle bevel up	1610	(82.7)
Venous needle rotated after insertion	685	(35.2)
Arterial needle gauge		
14	158	(8.1)
15	1610	(82.7)
16	160	(8.2)
17	12	(0.6)
Venous needle gauge		
14	157	(8.1)
15	1603	(82.4)
16	161	(8.3)
17	12	(0.6)

*AVF* Arteriovenous fistula

Other cannulation related complications included the number of cannulation attempts, of which there were three attempts in 68 episodes, four attempts in 16 episodes, and six attempts in one episode of cannulation. No patient required a new CVC to be inserted, an existing CVC was rarely used (0.3%), single needle dialysis did not occur, and there were rare instances of not proceeding with dialysis (0.7%). Extravasation (0.9%) and hematoma after dialysis (1.3%) rarely occurred. The average online Kt/V (*n* = 1084) was satisfactory at 1.38 (SD, 0.21). (Table 3).

**Table 3:** Cannula related complications

Variables	*n*	(%)
Number of cannulation attempts		
3	68	(3.5)
4	16	(0.8)
6	1	(0.1)
Used an existing CVC	6	(0.3)
Temporary insertion of a CVC	0	(0.0)
Single needle dialysis	0	(0.0)
No HD	13	(0.7)
Extravasation occurred	17	(0.9)
Hematoma	26	(1.3)
Mean Kt/V (n = 1084)		
Mean 1.38 SD (0.21)		

*n* number, % percentage, *CVC* Central venous catheter, *HD* Hemodialysis

### Successful cannulation and univariable analysis

After removing episodes of cannulation (*n* = 125, 6%) that had missing outcome information or those that could not be matched to a patient or nurse, 1946 episodes of cannulation remained for analysis. Successful cannulation at first attempt occurred in 95.6% (*n* = 1861) of cannulation episodes. The 85-miscannulation events occurred in 47 patients; therefore, a third (31.5%) of patients had at least one event of miscannulation.

Univariable patient characteristics associated with successful cannulation were older age of the access, fistula compared with graft, depth of access, and no areas of aneurysm. Univariable episodes of cannulation associated with success were nurse confidence, no stent, and absence of oedema, bruising, or hematoma. Variables associated with successful cannulation were non-use of ultrasound and arterial needle rotated after insertion. Univariable nurse characteristics associated with successful cannulation include male nurse and no postgraduate certificate in renal nursing. (Table 4).

**Table 4:** Univariable repeated measures logistic regression modelling for first-time cannulation success

Variable	Univariable OR (95% CI)	*p*-value
Patient variables		
Patient age	1.01 (1.00–1.02)	0.19
Patient gender		
Male	1.09 (0.68–1.76)	0.72
Female	1	
BMI	0.95 (0.95–1.01)	0.12
Steroids		
No	1.65 (0.61–4.44)	0.32
Yes	1	
PAI		
No	0.83 (0.32–2.11)	0.69
Yes	1	
Diabetes		
No	1.02 (0.64–1.63)	0.94
Yes	1	
PVD		
No	0.89 (0.53–1.49)	0.65
Yes	1	
Heart disease		
No	1.05 (0.66–1.68)	0.84
Yes	1	
Hypertension		
No	1.73 (0.91–3.29)	0.09
Yes	1	
Hypotension		
No	0.76 (0.32–1.81)	0.54
Yes	1	
Smoker		
No	0.58 (0.14–2.36)	0.44
Yes	1	
Log age of access	2.01 (1.65–2.46)	< 0.001
AV type		
Fistula	3.22 (1.79–5.80)	< 0.001
Graft	1	
AVF location		0.82
Brachio-cephalic	1.20 (0.45–3.19)	0.72
Radio-cephalic	1.35 (0.50–3.68)	0.59
Brachio-basilic	1	
Surgical revision <3mths		
No	1.71 (0.90–3.25)	0.10
Yes	1	
Depth of AVF		< 0.001
Superficial	15.0 (5.64–40.0)	< 0.001
Palpable	11.3 (4.34–29.4)	< 0.001
Non-palpable	1	
Vessel		
Straight	1.01 (0.59–1.72)	0.98
Zig-zag	1	
vBifurcation		0.21
Nil	0.58 (0.23–1.45)	0.71
Single	0.91 (0.33–2.49)	0.86
Multiple	1	
Aneurysm		
Yes	2.64 (1.34–5.18)	0.005
No	1	
Episodes of cannulation variables		
Log nurse confidence	1.19 (1.01–1.40)	0.03
Length of vessel	1.00 (0.94–1.06)	0.87
Bruit present – indicates stenosis		
No	0.71 (0.30–1.65)	0.42
Yes	1	
Stent in situ		
No	3.17 (1.32–7.61)	0.01
Yes	1	
Oedema present		
No	3.22 (1.40–7.38)	0.006
Yes	1	
Bruising present		
No	2.42 (1.39–4.19)	0.002
Yes	1	
Hematoma present		
No	2.46 (1.02–5.90)	0.04
Yes	1	
AVF soft with tendency for extravasation		
No	1.32 (0.74–2.36)	0.35
Yes	1	
Tourniquet use		
No	1.25 (0.76–2.07)	0.37
Yes	1	
Ultrasound use		
No	13.9 (8.18–23.6)	< 0.001
Yes	1	
Standard needle length		
No	2.56 (0.35–18.8)	0.36
Yes	1	
Arterial needle antegrade insertion		
No	1.76 (0.92–3.79)	0.09
Yes	1	
Arterial needle bevel up		
No	0.68 (0.40–1.14)	0.14
Yes	1	
Arterial needle rotated after insertion		
No	0.57 (0.36–0.91)	0.02
Yes	1	
Venous needle bevel up		
No	0.64 (0.37–1.10)	0.10
Yes	1	
Venous needle rotated after insertion		
No	0.74 (0.45–1.23)	0.25
Yes	1	
Arterial needle gauge		0.66
14	2.78 (0.30–25.7)	0.37
15	2.03 (0.26–16.0)	0.50
16	1.52 (0.17–13.6)	0.70
17	1	
Venous needle gauge		0.10
14	7.65 (1.40–41.6)	0.02
15	4.58 (1.12–18.8)	0.03
16	3.39 (0.71–16.1)	0.13
17	1	
Nurse variables		
Nurse age	0.99 (0.96–1.02)	0.41
Years as a RN	0.98 (0.96–1.00)	0.09
Years as HD nurse	0.98 (0.96–1.01)	0.29
Gender		
Male	4.88 (1.42–16.7)	0.01
Female	1	
Employment status		
Fulltime	1.32 (0.83–2.09)	0.25
Part time/casual	1	
Job Title		
RN	1.16 (0.66–2.02)	0.61
CN / SDN	1	
Highest education		
BN/RN	1.13 (0.66–1.94)	0.66
Graduate or higher	1	
Postgrad in renal nursing		
No	1.84 (1.15–2.93)	0.01
Yes	1	

*OR* Odds ratio, *CI* Confidence interval, *BMI* Body mass index, *PAI* Platelet aggregate inhibitor, *PVD* Peripheral vascular disease, *AV* Arteriovenous, *AVF* Arteriovenous fistula, *RN* Registered nurse, *HD* Hemodialysis, *CN* Clinical nurse, *SDN* Staff development nurse, *BN* Bachelors of nursing

There were no missed cannulation from the following variables; therefore, they were removed from the model: AVG location lower arm, immunosuppressant, anticoagulant, local anaesthetic, and cannulation technique

Current stenosis and bruit were highly correlated; therefore, removed current stenosis from the model

No male nurse had ‘post-graduate certificate in renal nursing’; therefore, ‘male nurse’ was removed from the model

### Multivariable analysis

In the multivariable patient access model, the variables significantly associated with successful cannulation were: older age of the access, a fistula compared with a graft, and absence of a stent in the fistula / graft. Episodes of cannulation variables significantly associated with successful cannulation were non-use of ultrasound and non-use of a tourniquet. The only nurse variable to be significantly associated with successful cannulation was non-completion of a postgraduate certificate in renal nursing. (Table 5).

**Table 5:** Multivariable repeated measures logistic regression modelling for first-time cannulation success

Variable	Multivariable OR (95% CI)	*p*-value
Patient variables		
Log age of access^a^	1.68 (1.32–2.14)	< 0.001
AV type		
Fistula	4.38 (1.89–10.1)	0.001
Graft	1	
Episodes of cannulation		
Stent in situ		
No	3.37 (1.39–8.19)	0.007
Yes	1	
Did not use ultrasound	13.7 (6.53–28.6)	< 0.001
	1	
Did not use tourniquet	2.32 (1.15–4.66)	0.02
	1	
Nurse variables		
Postgrad in renal nursing		
No	2.27 (1.31–3.93)	0.004
Yes	1	

*OR* Odds ratio, *CI* Confidence interval, *AV* Arteriovenous, *HD* Hemodialysis

^a^For every 1 year older, OR was 1.68 times more likely to be successful

## Discussion

Successful VA cannulation is important to minimise complications and maintain the longevity of an arteriovenous access. Furthermore, missed cannulation can be painful, result in fear and anxiety, and be burdensome for the patient. The major findings from this study reports a low miscannulation rate of 4.4%, and identifies multivariable characteristics associated with cannulation success that include: the older age of the access, AVF type access, absence of a stent, non-use of ultrasound and tourniquet, and non-completion of a postgraduate certificate in renal nursing.

Compared to our 4.4% rate of miscannulation, a recent cross-sectional study in 171 HD centres in Europe, the Middle East and South Africa [13] reported a much lower rate (1.1 to 1.8%) from over 10,000 cannulations. Interestingly, the authors reported a belief that the true prevalence of complications was lower than might be observed. In contrast, another study [3] reported a far higher percentage of patients with miscannulation (31%) in newly created VAs over a 6 month period; however, it is difficult to make comparisons with our study as the authors used the number of patients as the denominator rather than number of episodes of cannulation.

We report for the first time that older VA was associated with successful cannulation. This is not surprising, given that an older more developed access will have thickened vessel walls from repeated cannulation, increased diameter with maturation, and longer length of the vessel available for cannulation.

Interestingly, this study also found that cannulation was more likely to be successful when cannulating an AVF compared with an AVG. This is in complete contrast to a study by Van Loon et al. [3] who studied newly created VA in first-time patients with a six month follow up. Our cohort only had a small number of patients with an AVG, thus staff may have had less exposure and expertise in cannulating AVG compared with AVF.

To our knowledge, this is the first study to report an association between the absence of a stent with successful cannulation. The presence of a stent is associated with a more problematic VA with a tendency to stenosis. Stenosis would add a degree of complexity as the nurse would be placing the needle into a narrower section of the VA.

Surprisingly, we found that use of an ultrasound to assist with cannulation were more likely to be unsuccessful. This may reflect the complexity of the access and that a nurse may only use ultrasound if they considered the access difficult to cannulate. A recent study [23] reported the use of ultrasound as standard practice for central venous cannulation, evaluating AVF blood flow, and assessing for areas of thrombosis and aneurysms; however, ultrasound was not commonly used for real-time fistula cannulation. Another study [24] suggested that nursing uptake of the use of ultrasound was not widespread and skills using ultrasound were inconsistent. Other studies suggest more education is required to use ultrasound for cannulation [25] and more research is needed in this area [5]. Importantly, while 4.4% of missed cannulations may sound relatively few, we observed that one-third of patients (*n* = 47) had at least one episode of miscannulation over the study period, thus this remains a substantial problem to patients over their course of treatment.

Unexpectedly, we also found that not using a tourniquet was associated with cannulation success. This finding requires further research as the current nursing recommendations for the management of VA [21] suggests that tourniquets should be routinely used for cannulation. The guideline indicates that tourniquets help engorge the vein, making it more visible, and help to limit movement of the vein during cannulation. Although, the guideline also recognises that many expert cannulators repeatedly achieve successful cannulation without using a tourniquet.

We also found that nurses without a postgraduate certificate in renal nursing were more likely to be successful with cannulation. It is possible that nurses with the postgraduate certificate were more likely allocated to cannulate patients with more difficult access, as alluded to in a study by Parisotto and colleagues [13].

This study also found univariable characteristics associated with cannulation success that are worth mentioning. These include the depth of the access, areas of aneurysm, and if oedema, bruising, or hematoma were present before cannulation, and if the arterial needle was rotated after cannulation. We found an increased likelihood of successful cannulation if an access was palpable or superficial compared with one that was non-palpable.

Cannulation was also more likely to be successful if an access had areas of aneurysm. While areas with aneurysms are easier to cannulate and are less painful for the patient, repeated cannulations in the same area carries the risk of rupture, serious haemorrhage and may be fatal and should be avoided [7].

Not surprisingly, we found an association between the absence of oedema, bruising or hematoma with successful cannulation. In contrast to another study, [13] arterial needle rotation after insertion was more likely related to successful cannulation. This warrants further investigation. It is an unusual finding as it is generally thought that rotation of the needle results in additional trauma to the endothelial vascular wall, increasing the size of the puncture site, which may increase risk of bleeding during treatment and on withdrawal of the needle when dialysis is completed [13]. Nurses may rotate the needle if the needle was inserted bevel up in attempt to produce higher blood flow [13].

This study had both strengths and limitations. The strengths include the high number of episodes of cannulation from three different dialysis units over a seven-month period. We were able to adjust for patient characteristics, episodes of cannulation, and nurse characteristics in the analyses. Although we collected data over a seven-month period, documentation of episodes of cannulation were missed. Patients were able to ‘opt-out’ of the study and completion of the CRF by the nurses was voluntary. We also did not have the budget to use ultrasound to objectively confirm some measures such as bifurcation presence. They therefore may be subjective and recorded differently by the nurses, however these were routinely performed measurements used daily in clinical practice. A limitation of this study is not being able to adjust for time from VA creation to first cannulation, we did attempt to collect this data; however, we found it was poorly reported. Time from VA creation to first cannulation has now been added to the electronic patient database. There was also lack of data on VA diameter; though, we did collect age of VA and this correlates with larger artery size [8]. While our study assessed over a thousand episodes of cannulation, we may still be underpowered to detect significant differences in other variables that may be associated with cannulation success. Finally, the limitation inherent in the observational design means that conclusions can only be drawn about association, not causation.

## Conclusion

In conclusion, this study demonstrated a low rate of missed cannulations and the variables associated with successful VA access cannulation included; older VA, fistula compared with graft, and absence of stents. In addition, missed cannulation was associated with using ultrasound or a tourniquet, and completion of a postgraduate certificate in renal nursing. Further research is required on ultrasound-guided cannulation and to assess whether variables associated with successful cannulation identified in this study could be used to develop a VA cannulation complexity instrument. This is clinically relevant and important as once an access has been classified based on complexity, a patient can be matched to a suitably skilled competency-assessed nurse to perform the cannulation, thereby, minimising the risk of missed cannulation and improve patient care.

## Abbreviations

AVF: Arteriovenous fistula
AVG: Arteriovenous graft
CI: Confidence interval
CRF: Case report form
CVC: Central venous catheter
HD: Hemodialysis
HDF: Haemodiafiltration
HREC: Human Resource Ethics Committee
OR: Odd ratios
SD: Standard deviation
VA: Vascular access
